# “Real-world” effect of a peer counselor on breastfeeding outcomes in an urban prenatal clinic in the United States

**DOI:** 10.1186/s12884-020-03360-6

**Published:** 2020-11-07

**Authors:** Noelle G. Martinez, Angelina Strohbach, Fengling Hu, Lynn M. Yee

**Affiliations:** 1 Division of Maternal-Fetal Medicine, Department of Obstetrics and Gynecology, Northwestern University Feinberg School of Medicine, IL Chicago, USA; 2 Department of Family and Community Medicine, University of California San Francisco, California San Francisco, USA

**Keywords:** Breastfeeding continuation, Breastfeeding initiation, Health disparities, Peer counselor

## Abstract

**Background:**

One approach for improving breastfeeding support and alleviating breastfeeding disparities is the implementation of a clinic-based peer counselor. Our objective was to assess the “real life” effects of an autonomous peer counselor who provides tailored support to low-income, minority women based on individual needs rather than a pre-determined research protocol.

**Methods:**

This is a secondary analysis of a prospective cohort study of women receiving publicly funded prenatal care during the 6 months before and after introduction of a peer counselor in a single prenatal clinic. The peer counselor provided one-on-one antenatal and postpartum lactation support. Electronic medical record and survey data were collected. The primary outcome was breastfeeding continuation at 6 weeks postpartum. Secondary outcomes included breastfeeding comfort, confidence, and training satisfaction, any breastfeeding, and total breastfeeding duration. Bivariable and multivariable analyses were performed.

**Results:**

Peer counselor exposure was not associated with the primary outcome of continued breastfeeding at 6 weeks (55.6% with peer counselor versus 49.1% without; aOR 1.26, 95% CI 0.69–2.31). However, women with peer counselor exposure were more likely to be satisfied with breastfeeding training at the time of delivery (98.2% vs. 83.6%, *p* = 0.006) and were more likely to have performed any breastfeeding (89.8% vs. 78.9%, *p* = 0.04), which remained significant on multivariable analysis (aOR 2.85, 95% CI 1.11–7.32).

**Conclusions:**

Peer counselor interventions are a promising approach to increase breastfeeding initiation. Further research is required to inform the most efficacious approach while also allowing peer counselors to operate independently and in line with the specific needs of their clients.

## Background

Breastfeeding has well-established benefits for both mother and child, including maternal-child bonding, improved newborn immunity, and decreased maternal rates of ovarian and breast cancer [1–5]. As such, the World Health Organization (WHO), American College of Obstetricians and Gynecologists (ACOG), and American Academy of Pediatrics (AAP) recommend exclusive breastfeeding for the first six months of life [6–8]. Yet there are several well-documented barriers, both individual and societal in nature, which prevent mothers from executing their plans to breastfeed or to continue breastfeeding for the recommended duration. Such barriers include time constraints, embarrassment, lack of social support, maternal discomfort, factors related to latching, and return to work [9–11].

According to the CDC National Immunization Survey, 83.2% of U.S. children born in 2015 were ever breastfed, and 24.9% received exclusive breastfeeding through 6 months. Yet when these data are broken down by race, ethnicity, and socioeconomic status, stark disparities emerge. Among non-Hispanic white children, 85.9% are ever breastfed and 29.5% are breastfed exclusively through 6 months; however, among non-Hispanic black children, 69.4% are ever breastfed and just 17.2% are breastfed exclusively through 6 months. Similar disparities exist based on socioeconomic status, with children born to college-educated mothers or to higher income families being more likely to be breastfed than those born to high school-educated mothers or to lower income families. These disparities persist despite evidence that mothers across the socioeconomic gradient are aware of the nutritional, immunological, and psychosocial benefits of breastfeeding [9, 12, 13].

One approach for improving breastfeeding support and alleviating these disparities is the implementation of a clinic-based peer counselor (PC). The PC role is normally occupied by a woman of the same racial or socioeconomic background as the patient population. Typically, the counselor has herself breastfed, has completed training on a range of core topics, including infant development, nutrition, counseling techniques, and breastfeeding promotion, and seeks to help other women. PC interventions have consisted of a combination of prenatal counseling, inpatient visits following delivery, telephone support, and home visits. Evidence has associated peer lactation counseling with increased rates of breastfeeding initiation, duration, and exclusivity [14–24]. Although there have been numerous randomized control trials and other protocol-based studies of PCs, previous studies have focused on the efficacy of a particular PC protocol. Few studies have prospectively examined the “real life” effects of a fully autonomous PC who provides tailored support to women based on individual needs rather than a pre-determined research protocol. Therefore, the aim of this pre-post study was to evaluate the effects of introducing an autonomous lactation PC to a publicly funded prenatal clinic.

## Methods

### Design

This is a secondary analysis of data from the Navigating New Motherhood (NNM) study [25]. NNM was a postpartum patient navigation program located at a hospital-based women’s health clinic at a university tertiary care center in Chicago, IL. This clinic provides care to a diverse population of largely low-income, minority women seeking obstetric and gynecologic care. Women were eligible for enrollment if they were age 18 or greater, English-speaking, and received Medicaid-funded prenatal care at the study site. Upon enrollment, postpartum women were paired with a patient navigator who provided individualized support for retention in the medical system during this intense transition period. Full details of the original Navigating New Motherhood program have been previously reported [25].

### Setting

During the first six months (May 2015 – October 2015) of this 12-month NNM program, the clinic did not have a designated PC. During this period, patients received breastfeeding information and support largely from clinic providers (physicians and nurses) or from outside sources if sought by individual patients. No formal clinic-organized lactation support was provided. In November 2015, by chance occurring at the mid-point of the navigation study, a full-time PC was hired. The PC had been previously trained in breastfeeding support and underwent additional training via the inpatient nurse lactation counselors at this institution. She was supervised by the clinic Nurse Manager. The goals of integrating a PC into the clinic were to reduce logistical barriers to breastfeeding care and to allow for longitudinal breastfeeding support throughout the prenatal and postpartum periods, including a combination of outpatient and inpatient support. As is generally expected of PCs, this individual had personal breastfeeding experience, completed required training, and had a strong desire to help other women. She was available full-time during weekdays. She was a largely autonomous member of the clinic staff, using her own judgment to design her work style and individualize patient support. Although the NNM navigator worked with the PC to ensure postpartum patients were connected to the PC and were aware of her position, the PC functioned independently from the study.

Typically, the PC approached patients early in pregnancy, either at their first appointment or soon after presentation to prenatal care. At this first meeting, she would introduce herself to make patients aware of her presence and inform them of the lactation support available to them. During such meetings, she emphasized that she was a source of information and support whether or not the woman was interested in breastfeeding. Generally, her next follow-up was in third trimester, during which she checked in 2–3 times during routine prenatal care. Additional appointments separate from routine care were available upon request. The PC then followed up once more in the hospital in the immediate postpartum period. After delivery, contact was largely individualized to the patient’s needs, as some patients had no complications or challenges, whereas others required closer contact. Postpartum contact could continue for as long as the patient required support and was available to all patients regardless of whether she had interacted with the PC antenatally. The PC gave patients her phone number and texted with patients at her own discretion. While the PC initiated contact at standard times throughout pregnancy, she did not follow a pre-determined protocol and instead focused her efforts on providing individualized support in accordance with patient needs. Overall, the PC spent roughly 65% of her time seeing patients in clinic, 25% in the inpatient setting, and 10% providing text-based support. Of note, the PC performed all services without charges to patients or Medicaid. When additional breastfeeding issues arose that were beyond the PC scope of practice, she was trained to refer patients to the physician and nurse clinicians.

### Data Collection

Data from the NNM study included baseline surveys during the immediate postpartum hospitalization. Surveys covered a range of topics, including contraception and breastfeeding preferences, as well as maternal demographic data; surveys were both designed by the study team and adapted from the Pregnancy Risk Assessment Monitoring System items. Breastfeeding-specific questions included the length of intended breastfeeding, satisfaction with breastfeeding training, and breastfeeding comfort and confidence. Satisfaction, confidence, and comfort were asked using 5-point Likert scales, and responses were dichotomized for analyses such that any answers of “very” or “somewhat” positive were grouped and compared to all neutral or negative responses. Navigators accompanied mothers to their postpartum appointments and at that time conducted surveys. At this appointment, mothers were also given a Patient Health Questionnaire-9 (PHQ-9) to assess for postpartum depressive symptoms; postpartum depression was defined by a PHQ-9 score > 9, which corresponds to moderate depressive symptoms [26]. Lastly, navigators reached out by telephone at 6 months postpartum to follow up on contraception and breastfeeding outcomes. In addition to inquiring about breastfeeding continuation, verbally-administered surveys at that time queried reason for breastfeeding discontinuation, if the patient had discontinued breastfeeding. Answers were typically between 3 and 5 words and were transcribed verbatim; no further probes of answers were performed.

The primary outcome of this secondary analysis was self-reported breastfeeding continuation (exclusive or partial) at 6 weeks postpartum. This variable was captured via the six-week postpartum appointment survey. If the patient did not attend her postpartum appointment, the six-month follow-up call survey was used to determine length of breastfeeding continuation (*n* = 9). If no survey data were completed by the patient navigator at either time point, the patient chart was consulted to determine the duration of breastfeeding (*n* = 6).

Secondary outcomes included multiple patient-reported outcomes: breastfeeding intention (during postpartum hospitalization); breastfeeding training satisfaction, breastfeeding comfort, breastfeeding confidence (each assessed during postpartum hospitalization and 6 weeks postpartum); any breastfeeding (inclusive of any breastfeeding initiation at any time point, including postpartum hospitalization); length of breastfeeding duration (weeks); and breastfeeding continuation (exclusive or partial) at 6 months postpartum.

### Data Analysis

Outcomes were compared for women who delivered before versus after the introduction of the PC. The start point of PC introduction was designated as the first day that the PC started seeing patients in this practice, as the PC role was intended to support women of all gestational ages and all breastfeeding preferences, not just women who sustained a relationship with the PC spanning the perinatal period. Women who delivered after PC introduction were included in the PC cohort regardless of whether they had a documented visit with the PC, as this would most realistically capture real-world implementation conditions. Furthermore, the integration of the PC into clinic was considered the primary difference between study periods, and we hypothesized that PC presence as a new clinical staff member would positively affect breastfeeding support and culture in this clinical setting more broadly, even if women did not have individual appointments.

Bivariable analyses were performed using chi-square and Mann-Whitney U tests as appropriate. Multivariable logistic regression models accounting for cohort differences with a *p* < 0.10 were utilized to evaluate the independent relationship between presence of a full-time clinic PC and primary and secondary outcomes. As postpartum depression (based on depression screening at the postpartum visit) was the only significant difference between cohorts, it was the only characteristic that was included in regression models for outcomes measured at or after 6 weeks postpartum. Multivariable analysis was not performed for outcomes assessed during the postpartum hospitalization due to the lack of differences between cohorts. We additionally performed multivariable logistic regression models in the entire population retaining factors with *p* < 0.10 (maternal education, age, race and ethnicity, and neonatal intensive care unit admission) on bivariable analyses to identify patient factors associated with the primary outcome. Statistical analyses were performed using Excel (Microsoft Corporation, Redmond, WA) and Stata (StataCorp v15, *College Station, TX)*. All tests were two-sided and *p* < 0.05 defined statistical significance.

Additionally, limited qualitative analyses regarding reasons for breastfeeding discontinuation were performed by reviewing the transcribed answers regarding breastfeeding discontinuation. Participants’ brief answers were then classified by reason based on these direct answers to the single question and organized into themes. Themes were then quantified; in-depth qualitative analysis was not required because of the brevity of participant responses. The Institutional Review Board of Northwestern University approved this study, and all prospective NNM participants provided written, informed consent.

## Results

### Patient Enrollment and Patient Characteristics

During the NNM study period, 225 women were approached for program participation, and 96.9% (*n* = 218) enrolled. The cohort consisted of predominantly racial and ethnic minority women, and all women were publicly insured. Of the 218 enrolled patients, 45.4% (*n* = 99) were enrolled after the PC began working with patients. There were no significant differences between cohorts in terms of demographic or employment characteristics. There were no differences in clinical characteristics, with the exception of postpartum depression, which was marginally more frequent among women enrolled after introduction of the PC (25.3% with PC vs. 18.6% without PC, *p* = 0.05). The authors were unable to identify a clear reason for this difference. (Table 1)

**Table 1:** Patient characteristics before and after introduction of a peer counselor

**Characteristic**	**No PC(*n* = 119)**	**PC(*n* = 99)**	***p*-value**
Age, years	29.0 (± 5.1)	28.7 (± 5.2)	0.59
Pregravid BMI, kg/m^2^	30.4 (± 7.5)	29.5 (± 8.5)	0.32
Race/ethnicity			
Non-Hispanic White	16 (13.5)	12 (12.1)	0.28
Non-Hispanic Black	56 (47.1)	52 (52.5)	
White Hispanic	37 (31.1)	29 (29.3)	
Black Hispanic	5 (4.2)	0 (0.0)	
Asian	4 (3.4)	6 (6.1)	
Other	1 (0.8)	0 (0.0)	
Married	32 (27.1)	35 (35.7)	0.17
Multiparous	79 (66.4)	74 (74.8)	0.18
Some college education or greater	80 (67.8)	70 (72.9)	0.42
Employed for wages	50 (42.7)	34 (34.3)	0.21
Maternity Leave	37 (74.0)	25 (73.5)	0.96
Intended pregnancy	40 (33.6)	41 (41.4)	0.24
Antenatal depression^a^	19 (16.8)	14 (15.4)	0.78
Postpartum depression^b^	4 (4.0)	9 (11.7)	0.05
Spontaneous vaginal delivery	78 (65.6)	64 (64.7)	0.89
Infant NICU admission	22 (18.6)	25 (25.3)	0.24

Data displayed as n (%) or mean (± SD). *BMI* body mass index; *NICU* neonatal intensive care unit

^a^Defined by PHQ-9 > 9 prior to delivery

^b^Defined by PHQ-9 > 9 at 6-week postpartum appointment

### Breastfeeding Training

Within the no PC cohort, 56.2% reported receiving some form of lactation training, with most common sources being a physician (*n* = 28), an outside PC including from WIC (*n* = 29), or a nurse (*n* = 13). Of the cohort enrolled during the presence of a full-time PC, 81.4% received at least one formal consult session from the PC, and an additional 10.1% received training from other providers, with the most common being nurses (*n* = 7). Women in the PC cohort were more likely to be satisfied with breastfeeding training (98.2% vs. 83.6%, *p* = 0.006) when queried during the postpartum hospitalization. However, women in the PC cohort did not differ with regard to satisfaction with breastfeeding training when queried at six weeks postpartum (94.1% vs. 93.5%, *p* = 0.88) (Table 2).

**Table 2:** Breastfeeding outcomes associated with Introduction of clinic-based peer counselor

	Bivariable analysis			Multivariable analysis^a^
	No PC (*n* = 119)	PC (*n* = 99)	*p*-value	aOR (95% CI)
**Breastfeeding Training Satisfaction**^b^				
Postpartum hospitalization	56 (83.6)	55 (98.2)	0.006	
6 weeks postpartum	72 (93.5)	64 (94.1)	0.88	1.35 (0.31–5.93)
**Breastfeeding Intention**				
Intention to breastfeed	96 (80.7)	89 (89.9)	0.06	
**Breastfeeding Initiation and Continuation**				
Any breastfeeding	86 (78.9)	79 (89.8)	0.04	2.85 (1.11–7.32)
Breastfeeding continuation at 6 weeks	54 (49.1)	50 (55.6)	0.31	1.26 (0.69–2.31)
Exclusive breastfeeding at 6 weeks	18 (16.8)	21 (24.1)	0.21	1.61 (0.78–3.35)
Breastfeeding continuation ≥ 6 months	16 (18.0)	17 (21.8)	0.54	1.47 (0.66–3.27)
**Breastfeeding Comfort**^c^				
Postpartum hospitalization	87 (74.4)	78 (78.8)	0.45	
6 weeks postpartum	79 (79.0)	64 (83.1)	0.49	1.75 (0.74–4.11)
**Breastfeeding Confidence**^d^				
Postpartum hospitalization	92 (78.0)	81 (81.8)	0.48	
6 weeks postpartum	81 (79.4)	64 (83.1)	0.53	1.43 (0.63–3.24)

Data displayed as n (%) or adjusted odds ratio (95% confidence interval)

^a^Adjusted for postpartum depression if outcome was measured at or after 6 weeks postpartum

^b^Satisfaction defined as “satisfied” or “very satisfied” on 5-point Likert scale

^c^Comfort defined as “comfortable” or “very comfortable” on 5-point Likert scale

^d^Confidence defined as “confident” or “very confident” on 5-point Likert scale

### Breastfeeding Intention

Intention to breastfeed (reported during postpartum hospitalization) was higher in the PC cohort compared to no PC cohort (89.9% v. 80.7%) but the difference was not significant (*p* = 0.06). Intention to breastfeed 6 months or longer, in accordance with WHO, ACOG, and AAP recommendations, was endorsed by 47.9% in the PC cohort and by 48.5% in the no PC cohort (Table 2).

### Breastfeeding Initiation and Continuation

Women in the PC cohort were more likely to have initiated any breastfeeding, including during the postpartum hospitalization (89.8% vs. 78.9%, *p* = 0.04), which remained significant on regression analysis adjusting for postpartum depression (aOR 2.85, 95% CI 1.11–7.32) (Table 2).

The primary outcome of continued breastfeeding at 6 weeks was not associated with PC introduction (55.6% PC vs. 49.1% no PC, *p* = 0.31; aOR 1.26, 95% CI 0.69–2.31), nor was exclusive breastfeeding at 6 weeks (Table 2). Similarly, breastfeeding continuation at six months postpartum did not differ between cohorts (21.8% vs. 18.0%, *p* = 0.54). Thus, aside from the difference in whether any breastfeeding occurred, the overall duration of breastfeeding did not differ by cohort.

### Breastfeeding Comfort and Confidence

There were no significant differences between PC and no PC cohorts regarding breastfeeding comfort or confidence during the postpartum hospitalization or at six weeks postpartum (Table 2).

### Factors Associated with Breastfeeding Continuation

In the total study population, after controlling for potentially confounding factors, the only demographic or clinical factor associated with continued breastfeeding at 6 weeks was having some maternal college education or greater (aOR 3.48, 95% CI 1.64–7.39) (Table 3). In addition, when accounting for maternal education, age, race and ethnicity, and neonatal intensive care unit admission, endorsing breastfeeding comfort and reporting breastfeeding confidence (aOR 7.70, 95% CI 2.74–21.62 and aOR 8.29, 95% CI 2.97–23.08, respectively) were also associated with greater odds of continued breastfeeding at 6 weeks.

**Table 3:** Factors associated with continued breastfeeding at 6 weeks in the total cohort

	Bivariable analyses			Multivariable analysis*
Clinical or demographic factor	Not Breastfeeding (*n* = 96)	Breastfeeding (*n* = 104)	*p*-value	aOR (95% CI)
Age, years	27.9 (± 4.9)	30.1 (± 5.0)	0.004	1.05 (0.99–1.12)
Pregravid BMI, kg/m^2^	30.5 (± 7.3)	29.6 (± 8.5)	0.39	
Race/ethnicity Non-Hispanic White Non-Hispanic Black White Hispanic Black Hispanic Asian Other	11 (11.5) 50 (52.1) 32 (33.3) 2 (2.1) 0 (0.0) 1 (1.0)	13 (12.5) 50 (48.1) 29 (27.9) 2 (1.9) 10 (9.6) 0 (0.0)	0.05	(ref) 1.19 (0.45–3.15) 1.37 (0.48–3.95) 1.38 (0.14–13.75) -- --
Married	25 (26.3)	38 (36.9)	0.11	--
Multiparous	66 (68.8)	71 (68.3)	0.94	--
Some college education or greater	56 (60.2)	88 (85.4)	< 0.001	3.48 (1.64–7.39)
Employed for wages Maternity leave	38 (40.0) 28 (73.7)	40 (38.8) 29 (72.5)	0.87 0.91	--
Intended pregnancy	38 (39.6)	39 (37.5)	0.76	--
Antenatal depression	15 (16.7)	17 (17.4)	0.90	--
Postpartum depression	6 (7.1)	7 (7.7)	0.89	--
Spontaneous vaginal delivery	64 (66.7)	63 (60.6)	0.37	--
Infant NICU admission	26 (27.1)	18 (17.3)	0.095	0.48 (0.22–1.06)

Data displayed as *n* (%) or mean (± SD). *BMI* body mass index; *NICU* neonatal intensive care unit

*Multivariable model retained all factors associated with breastfeeding with *p* < 0.10 on bivariable analyses

### Breastfeeding Discontinuation

The most common reasons for breastfeeding cessation were related to perceived milk supply issues (*n* = 39), the time-consuming nature of breastfeeding (*n* = 20), and latch issues (*n* = 19). Other reasons included challenges with pain or bleeding, concern over maternal medication risks, and feeling emotionally overwhelmed (Table 4).

**Table 4:** Stated reasons for discontinuing breastfeeding (*n* = 96)

Reason	No Full-time PC (*n* = 47)	Full-time PC (*n* = 49)
Decreased milk supply	20 (42.6%)	19 (38.8%)
Time-consuming	12 (25.5%)	8 (16.3%)
Latch difficulties	7 (14.9%)	12 (25.5%)
Maternal health factors (i.e. taking unsafe medication)	6 (12.8%)	6 (12.2%)
Overwhelmed	6 (12.8%)	4 (8.2%)
Pain/bleeding	2 (4.3%)	4 (8.2%)
Infant Health (i.e. allergy)	1 (2.1%)	3 (6.1%)

Data displayed as *n* (%)

## Discussion

Despite established maternal and infant benefits of breastfeeding, there are striking and persistent socioeconomic, racial, and ethnic disparities in breastfeeding rates in the United States [11, 27, 28]. One proposed approach to alleviating such disparities is through PC support [11, 29]. Previous studies have tested the effect of predetermined PC protocols to increase breastfeeding uptake and continuation among these populations, yet few studies to date have performed an effectiveness evaluation in which PCs function autonomously in a clinical setting. Our study achieved such a real-world simulation, while also achieving extensive data collection through presence of a patient navigator. In this study, introduction of a PC in a clinic serving publicly insured, largely racial and ethnic minority women was associated with improved breastfeeding training satisfaction in the immediate postpartum period, as well as increased breastfeeding initiation. However, introduction of a PC was not associated with improved breastfeeding continuation at 6 weeks or 6 months postpartum. Continued breastfeeding at 6 weeks postpartum was associated with greater maternal education as well as with patient report of greater breastfeeding comfort and confidence.

Our findings are consistent with national data about disparities in breastfeeding. CDC data show that low-income women, compared to all women, continue to have the lowest rates of breastfeeding initiation, with approximately 80% of women with a poverty income ratio < 200 ever breastfeeding [30]. Similarly, our pre-intervention data show that in a clinic serving low-income women with public insurance, 78.9% initiate breastfeeding. Our study also supports the previously demonstrated association between educational attainment and breastfeeding outcomes, as breastfeeding at 6 weeks postpartum was significantly associated with college education. In contrast to previous studies, PC introduction was not associated with sustained breastfeeding at six weeks or six months postpartum in this study. This, however, may be due to short study time frame, and perhaps the full effects of PC presence could not be appreciated; it is possible that more time would allow for more longitudinal patient-PC interactions, as well as a greater clinic-wide emphasis on breastfeeding support, leading to more robust positive outcomes. Nevertheless, a full-time PC was associated with increased rates of any breastfeeding from 78.9–89.8%. This improvement aligns with the Healthy People 2020 target of increasing the proportion of infants who are ever breastfed from 74.0% in 2006 to 81.9% in 2020 [31]. Our finding also aligns with other studies showing an increase of 10–15% in rates of any breastfeeding among those receiving peer counseling in urban settings compared to control groups.[18, 21, 32].

Our study reinforces the association between higher educational attainment and breastfeeding outcomes, yet it also highlights the need for further research on the precise mechanism behind this association. Such research may better identify the specific mechanisms driving this disparity and inform interventions targeted at health information communication, educational access, workplace policies, self-efficacy, or other unknown factors. Additionally, given that comfort and confidence are associated with breastfeeding continuation, but that in our study, PC support was not associated with improved comfort or confidence, future research can assess not only the individual ways in which a PC can enhance these feelings, but should also assess the more systemic or structural determinants of comfort and confidence. Previous authors have rightly advocated the importance of discussing sociocultural factors contributing to breastfeeding practices in African American women, such as historical and individual trauma, body image, and familial power structures, and we encourage continued efforts to elucidate and alleviate such factors [33–36].

A key strength of this study is that extensive data collection was performed independent of the intervention of interest, and the PC herself was not responsible for collecting data on breastfeeding outcomes. In addition, the PC was given autonomy over her priorities, schedule, and protocol. Therefore, this study mimicked “real-world” circumstances while allowing for rigorous program assessment. However, one important limitation is that the intervention only consisted of one PC and thus generalizability is limited. Furthermore, given that the PC operated independently, it is difficult to characterize her approach or determine which parts of her approach, including method of communication or degree of patient contact, were most effective or could be improved. Specifically, while this patient population overwhelmingly preferred text message as a form of communication, the benefits and drawbacks of text message-based lactation support provided postpartum could not be analyzed or explored further. Lastly, as this was a secondary analysis of a 1-year study, the sample size was constrained and thus we may be underpowered to detect less frequent outcomes or outcomes that require greater time before benefit is observed.

## Conclusions

Our data ultimately suggest that PC interventions are a promising approach for providing culturally competent, patient-centered education and counseling, and may be associated with clinical benefits in low-income, largely minority populations. Yet, such programs need careful implementation to achieve the same benefits as randomized trial settings. Future implementation and dissemination research is required to optimize the introduction of PCs into clinical contexts. Such work may focus on further optimizing PC interventions by assessing inpatient versus outpatient timing, as well as placement in an obstetric setting versus pediatric setting [20]. Patient-centered perspectives on the areas of greatest benefit, perceived optimal timing for contact, type of counselor experience, mode of education, and other perspectives must also be gathered from qualitative and mixed methods research.

Additionally, we acknowledge the importance of research and interventions which integrate community-level and systems-level factors alongside the individual-level factors which affect breastfeeding behaviors [29, 37]. We recognize breastfeeding as a complex manifestation of culture, health, and identity, and we encourage our colleagues, including those developing and implementing PC programs, to continue working to support breastfeeding in all women who desire it.

## Data Availability

The datasets generated and/or analyzed during the current study are not publicly available due to the privacy limitations but are available from the corresponding author on reasonable request.

## Abbreviations

NNM: Navigating New Motherhood
PC: Peer counselor
CDC: Centers for Disease Control and Prevention
WHO: World Health Organization
ACOG: American College of Obstetricians and Gynecologists
AAP: American Academy of Pediatrics

## References

[1] Godfrey JR, Lawrence RA (2010). Toward optimal health: the maternal benefits of breastfeeding. J Womens Health (Larchmt).

[2] Bernardo L, Horta CGV (2013). Short-term effects of breastfeeding: a systematic review of the benefits of breastfeeding and diarrhoea and pneumonia mortality.

[3] Binns C, Lee M, Low WY (2016). The Long-Term Public Health Benefits of Breastfeeding. Asia Pac J Public Health.

[4] Saarinen UM, Kajosaari M (1995). Breastfeeding as prophylaxis against atopic disease: prospective follow-up study until 17 years old. Lancet.

[5] Horta BL (2013). Long-term effects of breastfeeding: a systematic review.

[6] Gynecologists AC (2013). .o.O.a., *ACOG Committee Opinion No. 570: Breastfeeding in Underserved Women: Increasing Initiation and Continuation of Breastfeeding*. Obstet Gynecol.

[7] Organization WH *Health Topics: Breastfeeding*. 2018; Available from: http://www.who.int/topics/breastfeeding/en/.

[8] American Academy of Pediatrics (2012). Breastfeeding and the Use of Human Milk. Pediatrics.

[9] Guttman N, Zimmerman DR (2000). Low-income mothers’ views on breastfeeding. Soc Sci Med.

[10] Kimbro RT (2006). On-the-job moms: work and breastfeeding initiation and duration for a sample of low-income women. Matern Child Health J.

[11] Jones KM (2015). Racial and ethnic disparities in breastfeeding. Breastfeed Med.

[12] Alexander A, Dowling D, Furman L (2010). What do pregnant low-income women say about breastfeeding?. Breastfeed Med.

[13] Zimmerman DR, Guttman N (2001). Breast is best”: knowledge among low-income mothers is not enough. J Hum Lact.

[14] Arlotti JP (1998). Breastfeeding among low-income women with and without peer support. J Community Health Nurs.

[15] Kistin N, Abramson R, Dublin P (1994). Effect of peer counselors on breastfeeding initiation, exclusivity, and duration among low-income urban women. J Hum Lact.

[16] Pugh LC (2002). Breastfeeding duration, costs, and benefits of a support program for low-income breastfeeding women. Birth.

[17] Anderson AK (2005). A randomized trial assessing the efficacy of peer counseling on exclusive breastfeeding in a predominantly Latina low-income community. Arch Pediatr Adolesc Med.

[18] Chapman DJ (2004). Effectiveness of breastfeeding peer counseling in a low-income, predominantly Latina population: a randomized controlled trial. Arch Pediatr Adolesc Med.

[19] Merewood A (2006). The effect of peer counselors on breastfeeding rates in the neonatal intensive care unit: results of a randomized controlled trial. Arch Pediatr Adolesc Med.

[20] Patel S, Patel S (2016). The Effectiveness of Lactation Consultants and Lactation Counselors on Breastfeeding Outcomes. J Hum Lact.

[21] Wambach KA (2011). A randomized controlled trial of breastfeeding support and education for adolescent mothers. West J Nurs Res.

[22] Olson BH (2010). A quasi-experimental evaluation of a breastfeeding support program for low income women in Michigan. Matern Child Health J.

[23] McCoy MB (2018). Associations Between Peer Counseling and Breastfeeding Initiation and Duration: An Analysis of Minnesota Participants in the Special Supplemental Nutrition Program for Women, Infants, and Children (WIC). Matern Child Health J.

[24] Chapman DJ (2010). Breastfeeding peer counseling: from efficacy through scale-up. J Hum Lact.

[25] Yee LM (2017). Using a Patient Navigator to Improve Postpartum Care in an Urban Women’s Health Clinic. Obstet Gynecol.

[26] Kroenke K, Spitzer RL, Williams JB (2001). The PHQ-9: validity of a brief depression severity measure. J Gen Intern Med.

[27] McKinney CO (2016). Racial and Ethnic Differences in Breastfeeding. Pediatrics.

[28] Prevention, Centers for Disease, C. and (2006). Racial and socioeconomic disparities in breastfeeding–United States, 2004. MMWR Morb Mortal Wkly Rep.

[29] Reis-Reilly H, Fuller-Sankofa N, Tibbs C (2018). Breastfeeding in the Community: Addressing Disparities Through Policy, Systems, and Environmental Changes Interventions. J Hum Lact.

[30] Centers for Disease Control and Prevention. *Rates of Any and Exclusive Breastfeeding by Socio-demographics among Children Born in 2015*. 2017.

[31] (US) (2011). O.o.t.S.G.U.C.f.D.C.a.P.U.O.o.W.s.H., *The Surgeon General’s Call to Action to Support Breastfeeding.*.

[32] Gross SM (2009). The differential impact of WIC peer counseling programs on breastfeeding initiation across the state of Maryland. J Hum Lact.

[33] Gross TT (2015). WIC peer counselors’ perceptions of breastfeeding in African American women with lower incomes. J Hum Lact.

[34] Perez-Escamilla R, Sellen D (2015). Equity in breastfeeding: where do we go from here?. J Hum Lact.

[35] DeVane-Johnson S (2017). Integrative Literature Review of Factors Related to Breastfeeding in African American Women: Evidence for a Potential Paradigm Shift. J Hum Lact.

[36] Spencer B, Wambach K, Domain EW (2015). African American Women’s Breastfeeding Experiences: Cultural, Personal, and Political Voices. Qual Health Res.

[37] Bentley ME, Dee DL, Jensen JL (2003). Breastfeeding among low income, African-American women: power, beliefs and decision making. J Nutr.

